# Special Issue “Application Progress and Insights of Nanoporous Materials”

**DOI:** 10.3390/ijms24097718

**Published:** 2023-04-23

**Authors:** Daniil Eurov, Dmitry Kurdyukov

**Affiliations:** Ioffe Institute, 195256 St. Petersburg, Russia; kurd.gvg@mail.ioffe.ru

Application Progress and Insights of Nanoporous Materials is an open Special Issue in *International Journal of Molecular Sciences*, which aims to publish original research and review papers concerning fundamental and applied aspects of nanoporous materials of various composition and morphology, investigation of their properties and underlying mechanisms for fabrication of porous media with the desired characteristics. Framing the design of novel functional and smart materials and their applications is also encouraged. 

Nanoporous materials, as an important subclass of nanomaterials, are of great interest in various areas of science and technology, including materials science, biomedicine and energy [1]. Nanoporous materials can be defined as crystalline or amorphous solids, which consist of an inorganic or organic framework supporting a nanoporous (with the size of pores less than 100 nm) structure. Nanoporosity leads to low density, large specific surface area, feasibility of surface functionalization, high load capacity and endows the materials with a range of novel properties in the chemical, physical, mechanical, thermal, electrical and acoustical fields [2]. 

Zeolites, porous carbon, inorganic porous phosphates, metal–organic frameworks, ceramics, silica, aerogels, pillared materials and various polymers are typical representations of nanoporous materials. The most important issue that one needs to address during the fabrication and/or functionalization of porous nanomaterials is the simultaneous control over multiple length scales, morphology, chemical composition and purity. These characteristics largely determine the potential applications of nanoporous solids as catalysts, physisorbents and chemisorbents, sensors, energy harvesting and storage devices, and therapeutic and diagnostic agents. For instance, three-dimensionally ordered macroporous structures with the internal nanostructured features, large surface area, the interconnectivity of the pores and the resulting fully accessible inner surface are attractive related to applications, including photonics, actuators and biomedical materials or devices [3].

The research interest in the section “Application Progress and Insights of Nanoporous Materials” includes finding new pathways for the design of novel smart nanoporous materials with enhanced porosity and functionality and their intended application in sensing, catalysis, sorption, energy conversion, electronic devices and biomedicine.

## References

[1] Xu Q. (2013). Nanoporous Materials Synthesis and Applications.

[2] Thommes M., Schlumberger C. (2021). Characterization of Nanoporous Materials. Annu. Rev. Chem. Biomol. Eng..

[3] Stein A. (2023). Achieving Functionality and Multifunctionality through Bulk and Interfacial Structuring of Colloidal-Crystal-Templated Materials. Langmuir.

